# Detection of nut allergens by mass spectrometry and structure analysis after food processing

**DOI:** 10.1186/2045-7022-3-S3-P56

**Published:** 2013-07-25

**Authors:** S Lepski, J Gerwin, J Brockmeyer

**Affiliations:** 1 Institute of Food Chemistry, WWU Muenster, Muenster, Germany

## Background

According to epidemiological studies, about 2-4% of the population in industrialized countries suffer from food allergies. The symptoms can range from minor skin rash to life threatening anaphylaxis and at present no effective therapy is available. Strict avoidance of the offending food is therefore mandatory for allergic individuals. Nut allergens are regarded highly problematic, because of the severe course of disease and the potential of cross-contamination during food processing. Due to a lack of sensitive and specific detection methods there are no certain threshold values.

Another important aspect is the impact of food processing on structure and allergenicity of nut proteins. Processing like roasting or extrusion can lead to reactions of allergens with other food ingredients and thereby change solubility, conformation and digestibility.

## Methods

Allergens from hazelnut, peanut, pistachio and walnut were extracted and digested by trypsin. The resulting peptides were analyzed by high performance liquid chromatography tandem mass spectrometry (HPLC-MS/MS). Protein identification was performed via peptide fragment fingerprinting (PFF) of designated marker proteins and database search.

Furthermore allergens were processed on the one hand by thermal treatment in presence of sugars and on the other hand by extrusion cooking together with maize. Structural and functional consequences of allergen modification were analyzed via electrophoretic techniques and mass spectrometry. The stability of hazelnut proteins against gastrointestinal degradation was tested by use of an *in vitro* model simulating gastric and duodenal digestion. Several hazelnut cultivars were characterized by two-dimensional polyacrylamid gel electrophoresis (2D-PAGE).

## Results

A mass spectrometric method for the detection of nut allergens was developed successfully. Additional matrix experiments showed that the analysis of allergens in chocolate and muesli is also possible. Food processing led to protein aggregation, Maillard products and changes in digestibility. The characterization of several hazelnut cultivars showed minor differences in protein content and composition.

## Conclusion

According to the results food processing has a strong impact on structure and digestibility of allergens. In addition, different nut cultivars show a certain degree of protein heterogeneity. All of this should be considered for the choice of suitable marker proteins for development of a detection method.

## Disclosure of interest

None declared.

